# CMA – a comprehensive Bioconductor package for supervised classification with high dimensional data

**DOI:** 10.1186/1471-2105-9-439

**Published:** 2008-10-16

**Authors:** M Slawski, M Daumer, A-L Boulesteix

**Affiliations:** 1 Sylvia Lawry Centre for Multiple Sclerosis Research, Hohenlindenerstr. 1, D-81677 Munich, Germany; 2 Department of Statistics, University of Munich, Ludwigstr. 33, D-80539 Munich, Germany

## Abstract

**Background:**

For the last eight years, microarray-based classification has been a major topic in statistics, bioinformatics and biomedicine research. Traditional methods often yield unsatisfactory results or may even be inapplicable in the so-called "*p *≫ *n*" setting where the number of predictors *p *by far exceeds the number of observations *n*, hence the term "ill-posed-problem". Careful model selection and evaluation satisfying accepted good-practice standards is a very complex task for statisticians without experience in this area or for scientists with limited statistical background. The multiplicity of available methods for class prediction based on high-dimensional data is an additional practical challenge for inexperienced researchers.

**Results:**

In this article, we introduce a new Bioconductor package called CMA (standing for "**C**lassification for **M**icro**A**rrays") for automatically performing variable selection, parameter tuning, classifier construction, and unbiased evaluation of the constructed classifiers using a large number of usual methods. Without much time and effort, users are provided with an overview of the unbiased accuracy of most top-performing classifiers. Furthermore, the standardized evaluation framework underlying CMA can also be beneficial in statistical research for comparison purposes, for instance if a new classifier has to be compared to existing approaches.

**Conclusion:**

CMA is a user-friendly comprehensive package for classifier construction and evaluation implementing most usual approaches. It is freely available from the Bioconductor website at .

## 1 Background

Conventional class prediction methods often yield poor results or may even be inapplicable in the context of high-dimensional data with more predictors than observations like microarray data. Microarray studies have thus stimulated the development of new approaches and motivated the adaptation of known traditional methods to the high-dimensional setting. Most of them are implemented in the R language [1] and freely available at  or from the bioinformatics platform . Meanwhile, the latter has established itself as a standard tool for analyzing various types of high-throughput genomic data including microarray data [2]. Throughout this article, the focus is on microarray data, but the presented package can be applied to any supervised classification problem involving a large number of continuous predictors such as, e.g. proteomic, metabolomic, or signal data. Model selection and evaluation of prediction rules turn out to be highly difficult in the *p *≫ *n *setting for several reasons: i) the hazard of overfitting, which is common to all prediction problems, is considerably increased by high dimensionality, ii) the usual evaluation scheme based on the splitting into learning and test data sets often applies only partially in the case of small samples, iii) modern classification techniques rely on the proper choice of hyperparameters whose optimization is highly computer-intensive, especially with high-dimensional data.

The multiplicity of available methods for class prediction based on high-dimensional data is an additional practical challenge for inexperienced researchers. Whereas logistic regression is well-established as the standard method to be used when analyzing classical data sets with much more observations than variables (*n *> *p*), there is no unique reference standard method for the *n *≪ *p *case. Moreover, the programs implementing well-known popular methods such as penalized logistic regression, nearest shrunken centroids [3], random forests [4], or partial least squares [5] are characterized by a high heterogeneity as far as input format, output format, and tuning procedures are concerned. Inexperienced users have thus to spend much effort understanding each of the programs and modifying the data formats, while potentially introducing severe errors which may considerably affect the final results. Furthermore, the users may overlook important tuning parameters or detail settings that sometimes noticeably contribute to the success of the classifier. Note that circumventing the problem of the multiplicity of methods by always using a single "favorite method" (usually the method in the user's expertise area or a method which has been identified as top-performing method in a seminal comparison study) potentially leads to poor results, especially when the considered method involves strong assumptions on the data structure.

From the difficulties outlined above, we conclude that careful model selection and evaluation satisfying accepted good-practice standards [6] is a very complex task for inexperienced users with limited statistical background. In this article, we introduce a new Bioconductor package called CMA (standing for "**C**lassification for **M**icro**A**rrays") for automatically performing variable selection, parameter tuning, classifier construction, and unbiased evaluation of the constructed classifiers. The primary goal of CMA is to enable statisticians with limited experience on high-dimensional class prediction or biologists and bioinformaticians with statistical background to achieve such a demanding task on their own. Without much time and effort, users are provided with an overview of the unbiased accuracy of most top-performing classifiers. Furthermore, the standardized evaluation framework underlying CMA involving variable selection and hyperparameter tuning can also be beneficial for comparison purposes, for instance if a new classifier has to be compared to existing approaches.

In a nutshell, CMA offers an interface to a total of more than twenty different classifiers, seven univariate and multivariate variable selection methods, different evaluation schemes (such as, e.g. cross-validation or bootstrap), and different measures of classification accuracy. A particular attention is devoted to preliminary variable selection and hyperparameter tuning, issues that are often neglected in current literature and software. More specifically, variable selection is always performed using the training data only, i.e. for each iteration successively in the case of cross-validation, following well-established good-practice guidelines [6-9]. Hyperparameter tuning is performed through an inner cross-validation loop, as usually recommended [10]. This feature is intended to prevent users from trying several hyperparameter values on their own and selecting the best results a posteriori, a strategy which would obviously lead to severe bias [11].

The CMA package is freely available from the Bioconductor website at 

### Overview of existing packages

The idea of an R interface for the integration of microarray-based classification methods is not new. The CMA package shows similarities to the Bioconductor package 'MLInterfaces' standing for "An interface to various machine learning methods" [12], see also the Bioconductor textbook [13] for a presentation of an older version. The MLInterfaces package includes numerous facilities such as the unified MLearn interface, the flexible learnerSchema design enabling the introduction of new procedures on the y, and the xvalSpec interface that allows arbitrary types of resampling and cross-validation to be employed. MLearn also returns the native R object from the learner for further interrogation. The package architecture of MLInterfaces is similar the CMA structure in the sense that wrapper functions are used to call classification methods from other packages.

However, CMA includes additional predefined features as far as variable selection, hyperparameter tuning, classifier evaluation and comparison are concerned. While the method xval is flexible for experienced users, it provides only cross-validation (including leave-one-out) as predefined option. As the CMA package also addresses inexperienced users, it includes the most common validation schemes in a standardized manner. In the current version of MLInterfaces, variable selection can also be carried out separately for each different learning set, but it does not seem to be a standard procedure. In the examples presented in the Bioconductor textbook [13], variable selection is only performed once using the complete sample. In contrast, CMA performs variable selection separately for each learning set by default. Further, CMA includes additional features for hyperparameter tuning, thus allowing an objective comparison of different class prediction methods. If tuning is ignored, simpler methods without (or with few) tuning parameters tend to perform seemingly better than more complex algorithms. CMA also implements additional measures of prediction accuracy and user-friendly visualization tools.

The package 'MCRestimate' [14,15] emphasizes very similar aspects as CMA, focussing on the estimation of misclassification rates and cross-validation for model selection and evaluation. It is (to our knowledge) the only Bioconductor package beside ours supporting hyperparameter tuning and the workflow is fully compatible with good practice standards. The advances of CMA compared to MCRestimate are summarized below. CMA includes much more classifiers (21 in the current version), which allows a comfortable extensive comparison without much effort. In particular, it provides an interface to recent machine learning methods, including two highly competitive boosting methods (tree-based and componentwise boosting). CMA also allows to pass arguments to the classifier, which may be useful in some cases, for instance to reduce the number of trees in a random forest for computational reasons. Furthermore, all the methods included in CMA support multi-class response variables, even the methods based on logistic regression (which can only be applied to binary response variables in MCRestimate). A very wide range of variable selection methods are available from CMA, e.g. fast implementations of important univariate test statistics including typical multi-class approach (Kruskal-Wallis/F-test). Moreover, CMA offers the possibility of constructing classifiers in a hybrid way: variable selection can be performed via the lasso and subsequently plugged into another algorithm. In addition to cross-validation, evaluation can be performed based on several most often used schemes such as bootstrap (and the associated '0.632' or '0.632+' estimators) or repeated subsampling. The definition of the learning sets can also be customized, which may be an advantage when, e.g. one wants to evaluate a classifier based on a single split learning/test data, as usual in the context of validation. CMA also includes additional accuracy measures which are commonly used in medical research. Convivial visualization tools are provided at the intention of either statisticians or practitioners. When several classifiers are run, the compare function produces ready-to-use tables listing different performance measures for several classifiers.

From the technical point of view, an additional advance is that CMA's implementation is fully organized in S4 classes, which bears advantages for both experienced users (who may easily incorporate their own functions) and inexperienced users (who have access to convenient visualization tools without entering much code). As a consequence, CMA has a clear inherent modular structure. The use of S4 classes is highly beneficial when adding new features, because it requires at most changes in one 'building block'. Furthermore, S4 classes offer the advantage of specifying the input data in manifold ways, depending on the user's needs. For example, the CMA users can enter their gene expression data as matrices, data frames combined with formulae, or ExpressionSets.

### Overview of class prediction with high-dimensional data and notations

#### Settings and Notation

The classification problem can be briefly outlined as follows. We have a predictor space X, here X ⊆ ℝ^*p *^(for instance, the predictors may be gene expression levels, but the scope of CMA is not limited to this case). The finite set of class labels is denoted as Y = {0, ..., *K *- 1}, with *K *standing for the total number of classes, and *P*(**x**, *y*) denotes the joint probability distribution on X×Y. We are given a finite sample *S *= {(**x**_1_, *y*_1_),...,(**x**_*n*_, *y*_*n*_)} of *n *predictor-class pairs. The considered task is to construct a decision function

$$\begin{array}{ll} \hat{f}: & X\to Y\\ & x\mapsto \hat{f}(x) \end{array}$$

such that the *generalization error*

(1) $$R[f]=E_{P}[L(\hat{f}(x),y)]=\int_{X\times Y}L(y,\hat{f}(x))\;dP(x,y)$$

is minimized, where *L*(·,·) is a suitable loss function, usually taken to be the indicator loss (*L*(*u*, *v*) = 1 if *u *≠ *v*, *L*(*u*, *v*) = 0 otherwise). Other loss functions and performances measures are discussed extensively in section 3.1.5. The symbol ^ indicates that the function is estimated from the given sample *S*.

#### Estimation of the generalization error

**Figure 1:**
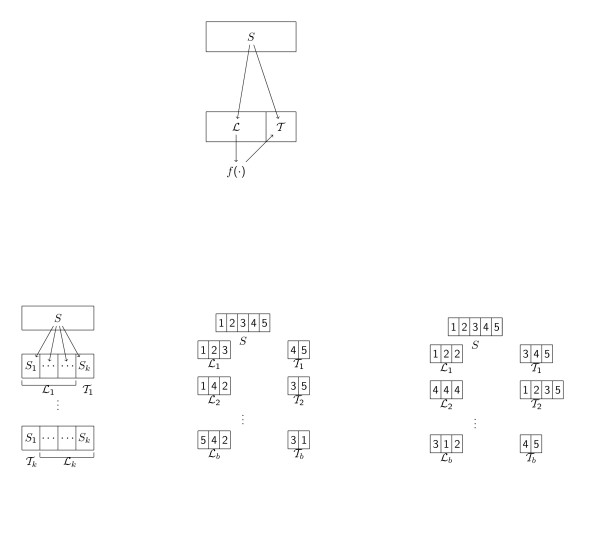
**Evaluation schemes**. The top panel illustrates the splitting into learning and test data sets. The whole sample *S *is split into a learning set ℒ and a test set T. The classifier *f*(·) is constructed using the learning set ℒ and subsequently applied to the test set T. The bottom panel displays schematically *k*-fold cross-validation (left), Monte-Carlo cross-validation with *n *= 5 and ntrain = 3 (middle), and bootstrap sampling (with replacement) with *n *= 5 and ntrain = 3 (right).

As we are only equipped with a finite sample *S *and the underlying distribution is unknown, approximations to Eq. (1) have to be found. The empirical counterpart to *R *[*f*]

(2) $$R_{\text{emp}}[f]=n^{-1}\sum_{i=1}^{n}L(y_{i},\hat{f}(x_{i}))$$

has a (usually large) negative bias, i.e. prediction error is underestimated. Moreover, choosing the best classifier based on Eq. (2) potentially leads to the selection of a classifier overfitting the sample *S *which may show poor performance on independent data. More details can be found in recent overview articles [16-18]. A better strategy consists of splitting *S *into distinct subsets ℒ (learning sample) and T (test sample) with the intention to separate model selection and model evaluation. The classifier $\hat{f}$(·) is constructed using ℒ only and evaluated using T only, as depicted in Figure 1 (top).

In microarray data, the sample size *n *is usually very small, leading to serious problems for both the construction of the classifier and the estimation of its prediction accuracy. Increasing the size of the learning set ($n_{L}$ → *n*) typically improves the constructed prediction rule $\hat{f}$(·), but decreases the reliability of its evaluation. Conversely, increasing the size of the test set ($n_{T}$ → *n*) improves the accuracy estimation, but leads to poor classifiers, since these are based on fewer observations. While a compromise can be found if the sample size is large enough, alternative designs are needed for the case of small sizes. The CMA package implements several approaches which are all based on the following scheme.

1. Generate *B *learning sets ${ℒ}_{b}$ (*b *= 1, ..., *B*) from *S *and define the corresponding test set as $T_{b}=S\backslash {ℒ}_{b}$

2. Obtain ${\hat{f}}_{b}$ (·) from ${ℒ}_{b}$, for *b *= 1, ..., *B*.

3. The quantity

(3) $$\hat{\epsilon }=\frac{1}{B}\sum_{b=1}^{B}\frac{1}{\left|T_{b}\right|}\sum_{i\in T_{b}}L(y_{i},\hat{f_{b}}(x_{i}))$$

is then used as an estimator of the error rate, where |·| stands for the cardinality of the considered set.

The underlying idea is to reduce the variance of the error estimator by averaging, in the spirit of the bagging principle introduced by Breiman [19]. The function GenerateLearningsets from the package CMA implements several methods for generating ${ℒ}_{b}$ and $T_{b}$ in step 1, which are described below.

LOOCV **Leaving-one-out cross-validation**

For the *b*-th iteration, $T_{b}$ consists of the *b*-th observation only. This is repeated for each observation in *S*, so that *B *= *n*.

CV *k*-**fold cross-validation **(method = "CV", fold, niter)

*S *is split into fold non-overlapping subsets of approximately equal size. For each iteration *b*, the *b*-th subset is used as $T_{b}$ and the union of the remaining subsets as ${ℒ}_{b}$, such that *B *= fold. Setting fold = n is equivalent to method = "LOOCV". For fold <*n*, the splitting is not uniquely determined. It is thus recommended to repeat the whole procedure niter times [16] (for instance niter = 5 or niter = 10) to partly average out random variations.

MCCV **Monte-Carlo-cross-validation **(method = "MCCV", fold, ntrain, niter)

Each of the *B *= niter learning sets of cardinality ntrain is drawn randomly from *S *without replacement. The argument ntrain specifies the number of observations to be included in each learning set.

boot **Bootstrap **(method = "bootstrap", ntrain, niter)

*B *= niter bootstrap samples (drawn with replacement) [20] of cardinality ntrain are used as learning sets. In practice, ntrain is usually set to the total sample size *n*.

A schematic representation of CV, MCCV and bootstrap sampling is provided in Figure 1 (bottom). "Stratified sampling" is possible by setting strat = TRUE. This implies that, in each learning set ${ℒ}_{b}$, the proportion of the classes {0, ..., *K *- 1} is approximately the same as in *S*. This option is very useful (and sometimes even necessary) in order to guarantee that each class is sufficiently represented in each ${ℒ}_{b}$, in particular if there are classes of small size. For more details on the evaluation of classifiers, readers may refer to recent overview articles discussing the respective drawbacks and advantages of these methods in [16,17].

Note that, if one employs a method to impute missing values making use of class label information, imputation should be performed for each learning set separately. This procedure is not supported by CMA. Instead, we recommend to impute missing values before beginning the analyses with CMA using a package like 'impute' [21] that does not involve class label information.

**Figure 2:**
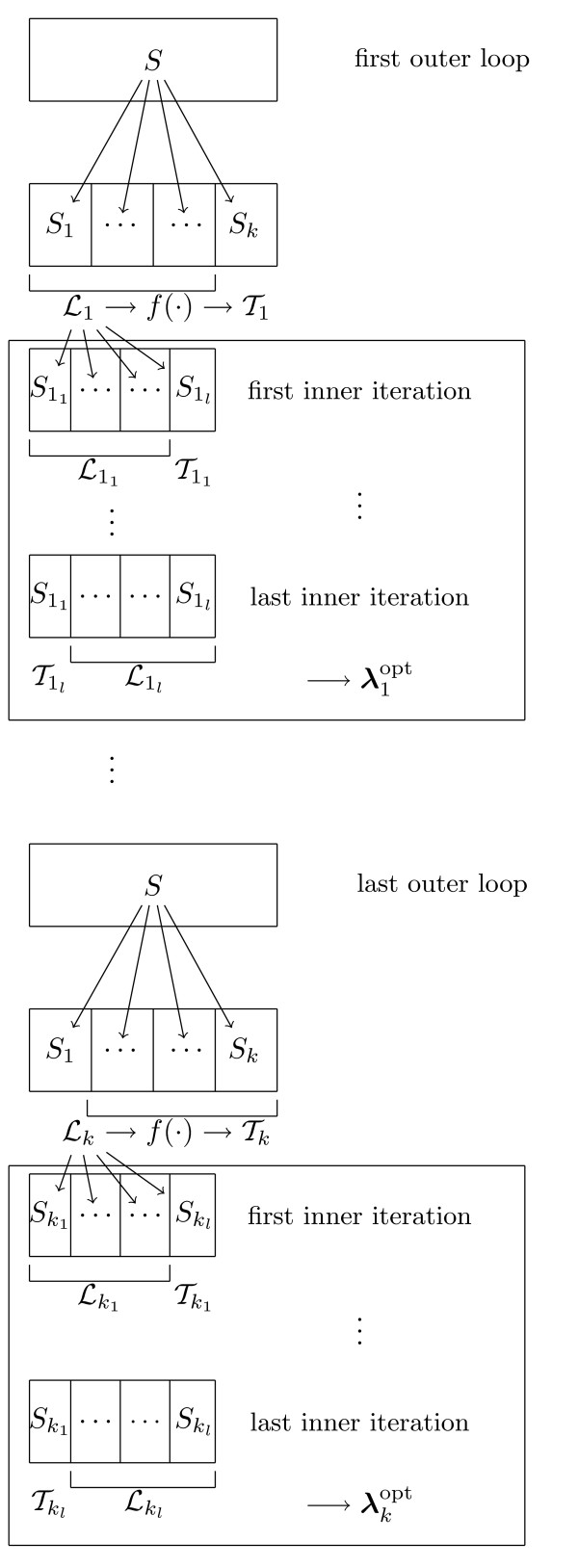
**Hyperparameter tuning**. Schematic display of nested cross-validation. In the procedure displayed above, *k*-fold cross-validation is used for evaluation purposes, whereas tuning is performed within each iteration using inner (*l*-fold) cross-validation.

In CMA, cross-validation is also used for hyperparameter tuning. The optimal value(s) of the method parameter(s) is(are) determined within an inner cross-validation, as commonly recommended [10,11]. If cross-validation is used for both tuning parameters and evaluating a classifiers, the whole procedure is denoted as nested cross-validation. See Figure 2 for a schematic representation and Section 3.1.4 for more details on hyperparameter tuning.

## 2 Implementation

The Bioconductor package CMA is user-friendly in the sense that (i) the methods automatically adapt to the data format provided by the user; (ii) convenient functions take over frequent tasks such as automatic visualization of results; (iii) reasonable default settings for hyperparameter tuning and other parameters requiring expert knowledge of particular classifiers are provided; (iv) it works with uniform data structures. To do so, CMA exploits the rich possibilities of object-oriented programming as realized by S4 classes of the methods package [22] which make it easy to incorporate new features into an existing framework. For instance, with some basic knowledge of the S4 class system (which is standard for bioconductor packages), users can easily embed new classification methods in addition to the 21 currently available in CMA. Moreover, the process of classifier building described in more detail in section 3.1.2 can either be partitioned into several transparent small steps (variable selection, hyperparameter tuning, etc) or executed by only one compact function call. The last feature is beneficial for users who are not very familiar with R commands.

## 3 Results

### 3.1 CMA features

#### 3.1.1 Overview

The package offers a uniform, user-friendly interface to a total of more than twenty classification methods (see Table 1) comprising classical approaches as well as more sophisticated methods. User-friendliness means that the input formats are the same for all implemented methods, that the user may choose between three different input formats and that the output is self-explicable and informative. The implementation is fully organized in S4 classes, thus making the extension of CMA very easy. In particular, own classification methods can easily be integrated if they return a proper object of class cloutput. In addition to the packages listed in Table 1, CMA only requires the package 'limma' for full functionality. For all other features, no code of foreign packages is used. Like most R packages, CMA is more flexible than, e.g., web-based tools. Experienced users can easily modify the programs or add new methods.

**Table 1:** Overview of the classification methods in CMA.

**Method name**	**CMA function name**	**Package**	**Reference**
Componentwise boosting	compBoostCMA	CMA	[39]
Diagonal discriminant analysis	dldaCMA	CMA	[56]
Elastic net	ElasticNetCMA	'glmpath'	[29]
Fisher's discriminant analysis	fdaCMA	CMA	[24]
Flexible discriminant analysis	flexdaCMA	'mgcv'	[24]
Tree-based boosting	gbmCMA	'gbm'	[33]
*k*-nearest neighbors	knnCMA	'class'	[24]
Linear discriminant analysis *	ldaCMA	'MASS'	[56]
Lasso	LassoCMA	'glmpath'	[57]
Feed-forward neural networks	nnetCMA	'nnet'	[24]
Probalistic nearest neighbors	pknnCMA	CMA	-
Penalized logistic regression	plrCMA	CMA	[58]
Partial Least Squares ⋆ + *	pls_ldaCMA	'plsgenomics'	[5]
⋆ + logistic regression	pls_lrCMA	'plsgenomics'	[5]
⋆ + random forest	pls_rfCMA	'plsgenomics'	[5]
Probabilistic neural networks	pnnCMA	CMA	[59]
Quadratic discriminant analysis	qdaCMA	'MASS'	[56]
Random forest	rfCMA	'randomForest'	[4]
PAM	scdaCMA	CMA	[44]
Shrinkage discriminant analysis	shrinkldaCMA	CMA	-
Support vector machines	svmCMA	'e1071'	[60]

The first column gives the method name, whereas the name of the classifier in the CMA package is given in the second column. For each classifier, CMA uses either own code or code borrowed from another package, as specified in the third column.

Moreover, CMA automatically performs all important steps towards the construction and evaluation of classifiers. It can generate learning samples as explained in section 1, including the generation of stratified samples. Different schemes for generating learning sets and test sets are displayed schematically in Figure 1 (bottom). The method GeneSelection provides optional variable selection preceding classification for each iteration *b *= 1, ..., *B *separately, based on various ranking procedures, whereas the method tune carries out hyperparameter tuning for a *fixed *(sub-)set of variables. It can be performed in a fully automatic manner using pre-defined grids. Alternatively, it can be completely customized by the experienced user. Performance can be assessed using the method evaluation for several performance measures commonly used in practice. Comparison of the performance of several classifiers can be quickly tabulated and visualized using the method comparison. Moreover, estimations of conditional class probabilities for predicted observations are provided by most of the classifiers, with only a few exceptions. This is more informative than only returning class labels and allows a more precise comparison of different classifiers. Last but not least, most results can conveniently be summarized and visualized using pre-defined convenience methods as demonstrated in section 3.2. For example, plot, cloutput-method produces probability plots, also known as "voting plot", plot, genesel-method visualizes variable importance as derived from one of the ranking procedures via a barplot, roc, cloutput-method draws empirical ROC curves, toplist, genesel-method lists the most relevant variables, and summary, evaloutput-method makes a summary out of iteration- or observationwise performance measures.

#### 3.1.2 Classification methods

This subsection gives a brief summarizing overview of the classifiers implemented in CMA. We have tried to compose a balanced mixture of methods from several fields although we do not claim our selection to be representative, taking into account the large amount of literature on this subject. For more detailed information on the single methods, readers are referred to the references given in Table 1 and the references therein. All classifiers can be constructed using the CMA method classification, where the argument classifier specifies the classification method to be used.

##### Discriminant Analysis

Discriminant analysis is the (Bayes-)optimal classifier if the conditional distributions of the predictors given the classes are Gaussian. Diagonal, linear and quadratic discriminant analysis differ only by their assumptions for the (conditional) covariance matrices **Σ**_*k *_= Cov(**x**|*y *= *k*), *k *= 0, ..., *K *- 1.

(a) Diagonal linear discriminant analysis (classifier = "dldaCMA") assumes that the within-class covariance matrices **Σ**_*k *_are diagonal and equal for all classes, i.e. $\Sigma_{k}=\Sigma =\text{diag}(\sigma_{1}^{2},...,\sigma_{p}^{2})$, *k *= 1, ..., *K *- 1, thus requiring the estimation of only *p *covariance parameters.

(b) Linear discriminant analysis (classifier = "ldaCMA") assumes **Σ**_*k *_= **Σ**, *k *= 1, ..., *K *- 1 without further restrictions for **Σ **so that *p*(*p *+ 1)/2 parameters have to be estimated.

(c) Quadratic discriminant analysis (classifier = "qdaCMA") does not impose any particular restriction on **Σ**_*k*_, *k *= 1, ..., *K *- 1.

While (a) turns out to be still practicable for microarray data, linear and quadratic discriminant analysis are not competitive in this setting, at least not without dimension reduction or excessive variable selection (see below).

The so-called PAM method (standing for "Prediction Analysis for Microarrays"), which is also commonly denoted as "shrunken centroids discriminant analysis" can be viewed as a modification of diagonal discriminant analysis (also referred to as "naive Bayes" classifier) using univariate soft thresholding [23] to perform variable selection and yield stabilized estimates of the variance parameters (classifier = "scdaCMA").

Fisher's discriminant analysis (FDA) (classifier = "fdaCMA") has a different motivation, but can be shown to be equivalent to linear discriminant analysis under certain assumptions. It looks for projections **a**^**T**^**x **such that the ratio of between-class and within-class variance is maximized, leading to a linear decision function in a lower dimensional space. Flexible discriminant analysis (classifier = "flexdaCMA") can be interpreted as FDA in a higher-dimensional space generated by basis functions, also allowing nonlinear decision functions [24]. In CMA, the basis functions are given by penalized splines as implemented in the R package 'mgcv' [25].

Shrinkage discriminant analysis [26,27] (classifier = "shrinkldaCMA") tries to stabilize covariance estimation by shrinking the unrestricted covariance matrix from linear discriminant analysis to a more simply structured target covariance matrix, e.g. a diagonal matrix.

##### Partial Least Squares

Partial Least Squares is a dimension reduction method that looks for directions ${\left\{w_{r}\right\}}_{r=1}^{R}$ maximizing $\left|\text{Cov}(y,w_{r}^{T}x)\right|$ (*r *= 1, ..., *R*) subject to the constraints $w_{r}^{T}w_{r}=1$ and $w_{r}^{T}w_{s}=0$ for *r *≠ *s*, where R ≪ *p*. Instead of working with the original predictors, one then plugs the projections living in a lower dimensional space into other classification methods, for example linear discriminant analysis (classifier = "pls_ldaCMA"), logistic regression (classifier = "pls_lrCMA") or random forest (classifier = "pls_rfCMA"). See Boulesteix and Strimmer [9] for an overview of partial least squares applications to genomic data analysis.

##### Regularization and shrinkage methods

In both penalized logistic regression and support vector machines, $\hat{f}$(·) is constructed such that it minimizes an expression of the form

(4) $$\sum_{i=1}^{n}L(y_{i},f(x_{i}))+\lambda J[f],$$

where *L*(·,·) is a loss function as outlined above and *J *[*f*] is a regularizer preventing overfitting. The trade-off between the two terms is known as bias-variance trade-off and governed via the tuning parameter *λ*. For ℓ^2 ^penalized logistic regression (classifier = "plrCMA"), *f*(**x**) = **x**^**T**^***β ***is linear and depends only on the vector *β *of regression coefficients, *J *[*f*] is the ℓ^2 ^norm *J *[*f*] = ***β***^**T**^***β ***and *L*(·,·) is the negative log-likelihood of a binomial distribution. Setting $J[f]=|\beta |=\sum_{j=1}^{p}\left|\beta_{j}\right|$ yields the Lasso [28] (classifier = "LassoCMA"), while combining both regularizers yields the elastic net [29] (classifier = "ElasticNetCMA"). CMA also implements a multi-class version of ℓ^2 ^penalized logistic regression, replacing the binomial negative likelihood by its multinomial counterpart.

For Support Vector Machines (classifier = "svmCMA"), we have

$$f(x)=\sum_{i\in V}\alpha_{i}k(x,x_{i}),$$

where V ⊂ {1, ..., *n*} is the set of the so-called "support vectors", *α*_*i *_are coefficients and *k*(·,·) is a positive definite kernel. Frequently used kernels are the linear kernel ⟨·,·⟩, the polynomial kernel ⟨·,·⟩^*d *^or the Gaussian kernel *k*(**x**_*i*_, **x**_*j*_) = exp((**x**_*i *_- **x**_*j*_)^*T*^(**x**_*i *_- **x**_*j*_)/*σ*^2^). The function *J *[*f *] is given as $J[f]=\sum_{i\in V}\sum_{j\in V}\alpha_{i}\alpha_{j}k(x_{i},x_{j})$  and L(.,.) is the so-called hinge loss [30].

##### Random Forests

The random forest method [4] aggregates an ensemble of binary decision-tree classifiers [31] constructed based on bootstrap samples drawn from the learning set (classifier = "rfCMA"). The "**b**ootstrap **agg**regat**ing**" strategy (abbreviated as "bagging") turns out to be particularly successful in combination with unstable classifiers such as decision trees. In order to make the obtained trees even more different and thus increase their stability and to reduce the computation time, random forests have an additional feature. At each split, a subset of candidate predictors is selected out of the available predictors. The random forest method also performs implicit variable selection and can be used to assess variable importance (see section 3.1.3).

##### Boosting

Similarly to random forests, boosting is based on a weighted ensemble of "weak learners" for classification, i.e. *f*(·) = ∑*α*_*m*_*f*_weak_(·), where *α*_*m *_> 0 (*m *= 1, ..., *M*) are adequately chosen coefficients. The term weak learner which stems from the machine learning community [32], denotes a method with poor performance (but still significantly better performance than random guessing) and low complexity. Famous examples for weak learners are binary decision trees with few (one or two) splits or linear functions in one predictor which is termed componentwise boosting. Friedman [33] reformulates boosting as a functional gradient descent combined with appropriate loss functions. The CMA package implements decision tree-based (classifier = "gbmCMA") and componentwise (classifier = "compBoostCMA") boosting with exponential, binomial and squared loss in the two-class case, and multinomial loss in the multi-class case.

##### Feed-Forward Neural Networks

CMA implements one-hidden-layer feed-forward neural networks (classifier = "nnetCMA"). Starting with a vector of covariates **x**, one forms projections $a_{r}^{T}x$, *r *= 1, ..., *R*, that are then transformed using an activation function *h*(·), usually sigmoidal, in order to obtain a hidden layer consisting of units ${\left\{z_{r}=h(a_{r}^{T}x)\right\}}_{r=1}^{R}$ that are subsequently used for prediction. Training of neural networks tends to be rather complicated and unstable. For large *p*, CMA works in the space of "eigengenes", following the suggestion of [34] by applying the singular value decomposition [35] to the predictor matrix.

##### Probabilistic Neural Networks

Although termed "Neural Networks", probabilistic neural networks (classifier = "pnnCMA") are actually a Parzen-Windows type classifier [36] related to the nearest neighbors approach. For **x **∈ T from the test set and each class *k *= 0, ..., *K *- 1, one computes

$$\begin{array}{cc} w_{k}=n_{k}^{-1}\sum_{x_{i}\in ℒ}I(y_{i}=k)\cdot \exp({\left(x_{i}-x\right)}^{T}(x_{i}-x)/\sigma^{2}), & k=0,...,K-1 \end{array}$$

where *n*_*k *_denotes the number of observations from class *k *in the learning set and *σ*^2 ^> 0 is a parameter. The quotient ${\left\{w_{k}/\sum_{k=0}^{K-1}w_{k}\right\}}_{k=0}^{K-1}$ is then considered as an estimate of the class probability, for *k *= 0, ..., *K *- 1.

##### Nearest Neighbors and Probabilistic Nearest Neighbors

CMA implements one of the variants of the ordinary nearest neighbors approach using the euclidean distance as distance measure (classifier = "knnCMA") and another variant called "probabilistic" that additionally provides estimates for class probabilities by using distances as weights, however without a genuine underlying probability model (classifier = "pknnCMA"). Given a learning set ℒ and a test set T, respectively, the probabilistic nearest neighbors method determines for each element in ℒ the *k *> 1 nearest neighbors N⊂ℒ and then estimates class probabilities as

$$\begin{array}{ccc} P(y=k|x)=\frac{\exp(\beta \sum_{x_{i}\in N}-d(x,x_{i})I(y_{i}=k))}{\exp(1+\beta \sum_{x_{i}\in N}-d(x,x_{i})I(y_{i}=k))}, & k=0,...,K-1, & x\in T \end{array}$$

where *β *> 0 is a method parameter and *d*(·,·) a distance measure.

Note that users can easily incorporate their own classifiers into the CMA framework. To do this, they have to define a classifier with the same structure as those already implemented in CMA. For illustrative purposes, we consider a simple classifier assigning observations to two classes. The code defining this classifier is given below. Once the classifier is defined, it can be used in the method classification in place of the CMA classifiers enumerated above.

myclassifier <- function(X, y, learnind, hyperpar = 1) {

   Xlearn <- X [learnind, ,drop = F]; yearn <- y [learnind]

   Xtest <- X [-learnind, ,drop = F]; ytest <- y [-learnind]

   w <- hyperpar * t(ylearn %*% Xlearn)

   pred <- (sign(drop(Xtest %*% w))+1)/2

   new("cloutput", learnind = learnind, y = ylearn,

      yhat = pred, prob = NA,

      method="myclassifier", mode = "binary")

}

#### 3.1.3 Variable selection methods

This section addresses the variable ranking- and selection procedures available in CMA. We distinguish three types of methods: pure filter methods (f) based on parametric or nonparametric statistical tests not directly related to the prediction task, methods which rank variables according to their discriminatory power (r), and classification methods selecting sparse sets of variables that can be used for other classification methods in a hybrid way (s). The multi-class case is fully supported by all the methods. Methods that are defined for binary responses only are applied within a "one-vs-all" or "pairwise" scheme. The former means that for each class *k *= 0, ..., *K *- 1, one recodes the class label *y *into *K *pseudo class labels ${\tilde{y}}_{k}$ = *I*(*y* = *k*) for *k *= 0,..., *K *- 1, while the latter considers all $\left(\begin{array}{c} K\\ 2 \end{array}\right)$ possible pairs of classes successively. The variable selection procedure is run *K *times or $\left(\begin{array}{c} K\\ 2 \end{array}\right)$ times, respectively, and the same number of genes are selected for each run. The final subset of selected genes consists of the union of the subsets obtained in the different runs.

In the CMA package, variable selection can be performed (for each learning set separately) using the method geneselection, with the argument method specifying the procedure and the argument scheme indicating which scheme (one-vs-all or pairwise) should be used in the *K *> 2 case. The implemented methods are:

(f) ordinary two-sample t.test (method = "t.test")

(f) Welch modification of the t.test (method = "welch.test")

(f) Wilcoxon rank sum test (method = "wilcox.test")

(f) F test (method = "f.test")

(f) Kruskal-Wallis test (method = "kruskal.test")

(f) "moderated" t and F test, respectively, using the package 'limma' [37] (method = "limma")

(r) one-step Recursive Feature Elimination (RFE) in combination with the linear SVM [38] (method = "rfe")

(r) random forest variable importance measure [4] (method = "rf")

(s) Lasso [28] (method = "lasso")

(s) elastic net [29] (method = "elasticnet")

(s) componentwise boosting (method = "boosting") [39]

(f) ad-hoc "Golub" criterion [40]

Each method can be interpreted as a function I(·) on the set of predictor indices: I: {1,..., *p*} → ℝ^+ ^where I(·) increases with discriminating power. I(·) is the absolute value of the test statistic for the (f) methods and the absolute value of the corresponding regression coefficient for the (s)-methods, while the (r)-methods are already variable importance measures per definition. Predictor *j *is said to be more important than predictor *l *if I(*l*) <I(*j*). It should be noted that the variable ordering is not necessarily determined uniquely, especially for the (s)-methods where variable importances are non-zero for few predictors only and for the (f) methods based on ranks. After variable ranking, variable selection is then completed by choosing a suitable number of variables (as defined by the user) that should be used by the classifier. For the multi-class case with one-vs-all or pairwise schemes, one obtains *K *and $\left(\begin{array}{c} K\\ 2 \end{array}\right)$ separate rankings, respectively, and the union of them forms the set of predictor variables. We again emphasize that the variable importance assignment is based on learning data only, which means that the procedure is repeated for each learning/test set splitting successively.

Note that the method GeneSelection may be used to order any kind of variables, not only genes. For example, if one uses CMA to classify genes based on samples instead of classifying samples based on genes, the function GeneSelection is used to select samples instead of selecting genes in spite of its name.

#### 3.1.4 Hyperparameter tuning

The function tune of the CMA package implements inner cross-validation for hyperparameter tuning, as represented schematically in Figure 2. The following procedure is repeated for each learning set ${ℒ}_{b}$ defined by the argument learningsets. The learning set is partitioned into *l *subsets of approximately equal size. For different values of the hyperparameters, the error rate is estimated based on ${ℒ}_{b}$ within a *l*-fold cross-validation scheme. The hyperparameter values yielding the smallest cross-validated error rate are then selected and used for the construction of the classifier based on ${ℒ}_{b}$. Hence, the test data set $T_{b}$ is not used for hyperparameter tuning. This procedure is often denoted as inner or internal cross-validation, yielding a so-called nested cross-validation procedure if cross-validation is also used to evaluate the error rate of the classification method of interest.

Examples of tuning parameters for the classifiers included in CMA are given in Table 2. Note that one could also consider the number of genes as an hyperparameter to be tuned in nested cross-validation, although this is still not standard practice. We plan to do this extension in future work.

**Table 2:** Overview of hyperparameter tuning in CMA.

**Method**	**Name in CMA**	**Range**	**Signification**
gbmCMA	n.trees	1, 2,...	number of base learners (decision trees)
LassoCMA	norm.fraction	[0;1]	relative bound imposed on the ℓ^1 ^norm on the weight vector
knnCMA	k	1, 2,...,|ℒ|	number of nearest neighbours
nnetCMA	size	1, 2, ...	number of units in the hidden layer
scdaCMA	delta	ℝ^+^	shrinkage towards zero applied to the centroids
svmCMA	cost	ℝ^+^	cost: controls the violations of the margin of the hyperplane
	gamma	ℝ^+^	controls the width of the Gaussian kernel (if used)

The first column gives the method name, whereas the name of the hyperparameter in the CMA package is given in the second column. The third column gives the range of the parameter and the fourth column its signification.

It is crucial to perform hyperparameter tuning properly, for instance within an inner cross-validation as implemented in CMA. On the one hand, by using a complicated classifier involving many parameters without tuning them, one implicitly favors simpler classifiers which do not involve any hyperparameters. On the other hand, it would be completely incorrect to tune the parameters a posteriori, i.e. to try several values of the tuning parameters successively and to show only the best results [11]. Such a design would artificially favor complicated classifiers with many tuning parameters, and one would expect the obtained optimal classifier to generalize poorly on an independent validation data set. This problem is connected to Occam's Razor.

For example, let us consider diagonal discriminant analysis (DLDA), which is also known as Naive Bayes classifier due to its simplicity, and on the other hand a SVM with a Gaussian kernel, which depends on at least two hyperparameters: the first one is the width of the Gaussian kernel and the second one is the cost parameter that governs the amount of regularization. While there are rules of thumb for choosing the width of the Gaussian kernel, this does not apply to the cost. Setting the cost to an arbitrary value can cause both over- and underfitting. In contrast, DLDA does not overfit due to its simplicity, but may tend to underfit.

Note that the hyperparameter tuning procedure may yield sub-optimal values for the hyperparameters if used in combination with the gene selection procedure. That is because, in the present version of the CMA package, the set of genes remains fixed for all iterations of the inner cross-validation. This may affect the performance of the tuning procedure in the following way. Since in the inner cross-validation procedure variable selection is based on both the training sets and the test sets, the selected gene subset fits the test sets artificially well, which may affect the selection of the optimal hyperparameter values. For example, hyperparameter values corresponding to too complex models (for instance a too low penalty in penalized logistic regression) might yield small cross-validation error rates and get selected by the inner cross-validation procedure. Thus, using the tuning procedure in combination with gene selection tends to yield sub-optimal hyperparameter values. This may result in overestimated error rates in outer cross-validation, but not in "false positive research findings" [41], in the sense that in this case CMA will rather underestimate the association between predictors and response than find an association when there is none.

That is why we recommend to use the tuning procedure of CMA only with methods that do not require any preliminary variable selection. Note that most of the classification methods needing tuning do not require any preliminary variable selection (like, e.g., support vector machines, shrunken centroids discriminant analysis, penalized logistic regression). Hence, this recommendation is not very restrictive in practice. However, we plan to modify the tuning procedure of CMA in a future version in order to allow the combination of tuning and gene selection. This can be done by re-performing gene selection in each inner cross-validation iteration successively, as already correctly implemented in the existing package 'MCRestimate' [14,15].

#### 3.1.5 Performance measures

Once the classification step has been performed for all *B *iterations using the method classification, the method evaluation offers a variety of possibilities for evaluating the results. As accuracy measures, the user may choose among the following criteria.

##### • Misclassification rate

This is the simplest and most commonly used performance measure, corresponding to the indicator loss function in Eq. (1). From *B *iterations, one obtains a total of $\sum_{b}\left|T_{b}\right|$ predictions. It implies that, with most procedures, the class label of each predictor-class pair in the sample *S *is predicted several times. The method evaluation can be applied in two directions: one can compute the misclassification rate either iterationwise, i.e. for each iteration separately (scheme="iterationwise"), yielding ${\hat{\epsilon }}^{\text{iter}}={\left({\hat{\epsilon }}_{b}\right)}_{b=1}^{B}$ or observationwise, i.e. for each observation separately (scheme = "observationwise"), yielding ${\hat{\epsilon }}^{\text{obs}}={\left({\hat{\epsilon }}_{i}\right)}_{i=1}^{n}$.  The latter can be aggregated by classes which is useful in the frequent case where some classes can be discriminated better than the other. Furthermore, observationwise evaluation can help identifying outliers which are often characterized by high misclassification error rates. Although ${\hat{\epsilon }}^{\text{iter}}$ or ${\hat{\epsilon }}^{\text{obs}}$ can be further averaged, the whole vectors are preferred to their less informative average, in order to reflect uncertainty more appropriately. A second advantage is that graphical summaries in the form of boxplots can be obtained.

##### • Cost-based evaluation

Cost-based evaluation is a generalization of the misclassification error rate. The loss function is defined on the discrete set {0, ..., *K *- 1} × {0, ..., *K *- 1}, associating a specific cost to each possible combination of predicted and true classes. It can be represented as a matrix ***L ***= (*l*_*rs*_), *r*, *s *= 0,...,(*K *- 1) where *l*_*rs *_is the cost or loss caused by assigning an observation of class *r *to class *s*. A usual convention is *l*_*rr *_= 0 and *l*_*rs *_> 0 for *r *≠ *s*. As for the misclassification rate, both iteration- and observationwise evaluation are possible.

##### • Sensitivity, specificity and area under the curve (AUC)

These three performance measures are standard measures in medical diagnosis, see [18] for an overview. They are computed for binary classification only.

##### • Brier Score and average probability of correct classification

In classification settings, the Brier Score is defined as

$$n^{-1}\sum_{i=1}^{n}\sum_{k=0}^{K-1}{\left(I(y_{i}=k)-\hat{P}(y_{i}=k|x_{i})\right)}^{2},$$

where $\hat{P}$(*y *= *k*|***x***) stands for the estimated probability for class *k*, conditional on ***x***. Zero is the optimal value of the Brier Score.

A similar measure is the average probability of correct classification which is defined as

$$n^{-1}\sum_{i=1}^{n}\sum_{k=0}^{K-1}\left(I(y_{i}=k)\hat{P}(y_{i}=k|x\right),$$

and equals 1 in the optimal case. Both measures have the advantage that they are based on the continuous scale of probabilities, thus yielding more precise results. As a drawback, however, they cannot be applied to all classifiers but only to those associated with a probabilistic background (e.g. penalized regression). For other methods, they can either not be computed at all (e.g. nearest neighbors) or their application is questionable (e.g. support vector machines).

##### • 0.632 and 0.632+ estimators

The ordinary misclassification error rate estimates resulting from working with learning sets of size <*n *tend to overestimate the true prediction error. A simple correction proposed for learning sets generated from bootstrapping (argument method="bootstrap" in the function GenerateLearningsets) uses a convex combination of the re-substitution error -which has a bias in the other direction (weight: 0.368) and the bootstrap error estimation (weight: 0.632). A further refinement of this idea is the 0.632+ estimator [42] which is approximately unbiased and seems to be particularly appropriate in the case of overfitting classifiers.

The method compare can be used as a shortcut if several measures have to be computed for several classifiers. The function obsinfo can be used for outlier detection: given a vector of observationwise performance measures, it filters out observations for which the classifier fits poorly on average (i.e. high misclassification rate or low Brier Score, for example).

### 3.2 A real-life data example

#### 3.2.1 Application to the SRBCT data

This section gives a demonstration of the CMA package through an application to real life microarray data. It illustrates the typical workflow comprising learning set generation, variable selection, hyperparameter tuning, classifier training, and evaluation. The small blue round cell tumor data set was first analyzed by Khan et al [43] and is available from the R package 'pamr' [44]. It comprises *n *= 65 samples from four tumor classes and expression levels from *p *= 2308 genes. In general, good classification results can be obtained with this data set, even with relatively simple methods [45]. The main difficulty arises from the two classes with small size (8 and 12 observations, respectively).

CMA implements a large number of classifiers and variable selection methods. In this demonstrating example, we compare the performance of seven of them, which are representative of the CMA functionalities: i) diagonal linear discriminant without variable selection and tuning, ii) linear discriminant analysis with variable selection, iii) quadratic discriminant analysis with variable selection, iv) Partial Least Squares followed by linear discriminant analysis with tuning of the number of components, v) shrunken centroids discriminant analysis with tuning of the shrinkage parameter, vi) support vector machines with radial kernel without tuning (i.e. with the default parameter values of the package 'e1071'), and vii) support vector machines with radial kernel and with tuning of the cost parameter and the width of the Gaussian kernel.

We choose to work with stratified five-fold cross-validation, repeated ten times in order to achieve more stable results [16]. For linear discriminant analysis we decide to work with ten and for quadratic discriminant analysis with only two variables. These numbers are chosen arbitrarily without any deeper motivation, which we consider legitimate for the purpose of illustration. In practice, this choice should be given more attention. We start by preparing the data and generating learning sets:

> *data(khan)*

> *khanY <- khan [, 1]*

> *khanX <- as.matrix(khan [, -1])*

> *set.seed(27611)*

> *fiveCV10iter <- GenerateLearningsets(y = khanY, method = "CV", fold = 5, niter = 10, strat = TRUE)*

khanY is an *n*-vector of class labels coded as 1,2,3,4, for the number of transcripts. fiveCV10iter is an object of class learningsets that stores for each of the niter = 10 iterations which observations belong to the learning sets as generated by the chosen method specified through the arguments method, fold and strat. For reproducibility purposes, it is crucial to set the random seed.

As a preliminary step to classification, we then perform variable selection for those methods requiring it (linear and quadratic discriminant analysis). For illustrative purposes, we first try several variable selection methods and display a part of their results for comparison.

> *genesel_f <- GeneSelection(X = khanX, y = khanY, learningsets = fiveCV10iter, method = "f.test")*

> genesel_kru <- GeneSelection(X = khanX, y = khanY, learningsets = fiveCV10iter, method = "kruskal.test")

> genesel_lim <- GeneSelection(X = khanX, y = khanY, learningsets = fiveCV10iter, method = "limma")

> genesel_rf <- GeneSelection(X = khanX, y = khanY, learningsets = fiveCV10iter, method = "rf", seed = 100)

For comparing these four variable selection methods, one can now use the toplist method on the objects genesel_f, genesel_kru, genesel_wil, genesel_rf to show, e.g. their top-10 genes. Genes are referred to by their column index in X. The commands given below display the 10 top-ranking genes found based on the first learning set using each of the four methods.

> tab <- cbind(f.test = toplist(genesel_f, s = F) [, 1], kru.test = toplist(genesel_kru, s = F) [, 1], lim.test = toplist(genesel_lim, s = F) [, 1], rf.imp = toplist(genesel_rf, s = F) [, 1])

> rownames(tab) <- paste("top", 1:10, sep = ".")

> print(tab)

**Table 3:** 

	f.test	kru.test	lim.test	rf.imp
top.1	1954	1194	22	545
top.2	1389	545	26	1954
top.3	1003	1389	723	2050
top.4	129	2050	1897	1003
top.5	1955	1954	148	246
top.6	246	246	428	1389
top.7	1194	1003	1065	187
top.8	2050	554	11	554
top.9	2046	1708	735	2046
top.10	545	1158	62	1896

The object tab indicates how many methods selected each gene in the top-10 list. We observe a moderate overlap of the four lists, with some genes appearing in three out of four lists:

> table(drop(tab))

**Table 4:** 

11	22	26	62	129	148	187	246	428	545	554	723	735	1003	1065	1158
1	1	1	1	1	1	1	3	1	3	2	1	1	3	1	1
1194	1389	1708	1896	1897	1954	1955	2046	2050							
2	3	1	1	1	3	1	2	3							

We now turn to hyperparameter tuning, which is performed via nested cross-validation. For Partial Least Squares, we optimize the number of latent components *R *over the grid {1,..., 5}. For the nearest shrunken centroids approach, the shrinkage intensity Δ is optimized over the grid {0.1, 0.25, 0.5, 1, 2, 5} (default). For the SVM with Gaussian kernel, one has to tune two hyperparameters: the cost for violating the margin in the primal formulation of the SVM and the width of the Gaussian kernel (see section tuning) which are optimized over {0.1, 1, 5, 10, 50, 100, 500} × {1/(4*p*), 1/(2*p*), 1/*p*, 2/*p*, 4/*p*} (also default).

> tune_pls <- tune(X = khanX, y = khanY, learningsets = fiveCV10iter, classifier = pls_ldaCMA, grids = list(comp = 1:5))

> tune_scda <- tune(X = khanX, y = khanY, learningsets = fiveCV10iter, classifier = scdaCMA, grids = list( ))

> tune_svm <- tune(X = khanX, y = khanY, learningsets = fiveCV10iter, classifier = svmCMA, grids = list( ), kernel = "radial")

In the second and third function calls to tune, the argument grids( ) is an empty list, which means that the default settings are used. The objects created in the steps described above are now passed to the function classification. The object genesel_f is passed to the classifiers ldaCMA and qdaCMA, since the F-test is the standard approach for variable selection in the multi-class setting. The argument nbgene indicates that only the "best" nbgene genes are used, where "best" is understood in terms of the F ratio.

> class_dlda <- classification(X = khanX, y = khanY, learningsets = fiveCV10iter, classifier = dldaCMA)

> class_lda <- classification(X = khanX, y = khanY, learningsets = fiveCV10iter, classifier = ldaCMA, genesel = genesel_f, nbgene = 10)

> class_qda <- classification(X = khanX, y = khanY, learningsets = fiveCV10iter, classifier = qdaCMA, genesel = genesel_f, nbgene = 2)

> class_plsda <- classification(X = khanX, y = khanY, learningsets = fiveCV10iter, classifier = pls_ldaCMA, tuneres = tune_pls)

> class_scda <- classification(X = khanX, y = khanY, learningsets = fiveCV10iter, classifier = scdaCMA, tuneres = tune_scda)

> class_svm <- classification(X = khanX, y = khanY, learningsets = fiveCV10iter, classifier = svmCMA, kernel = "radial")

> class_svm_t <- classification(X = khanX, y = khanY, learningsets = fiveCV10iter, classifier = svmCMA, tuneres = tune_svm, kernel = "radial")

**Figure 3:**
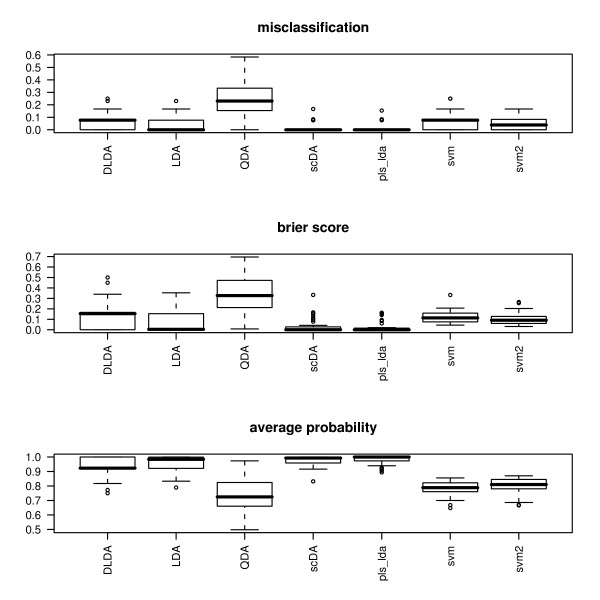
**Classification accuracy with Khan's SRBCT data**. Boxplots representing the misclassification rate (top), the Brier score (middle), and the average probability of correct classification (bottom) for Khan's SRBCT data, using seven classifiers: diagonal linear discriminant analysis, linear discriminant analysis, quadratic discriminant analysis, shrunken centroids discriminant analysis (PAM), PLS followed by linear discriminant analysis, SVM without tuning, and SVM with tuning.

The classification results can now be visualized using the function comparison, which takes a list of classifier outputs as input. For instance, the results may be tabulated and visualized in the form of boxplots, as displayed in Figure 3:

> classifierlist <- list(class_dlda, class_lda, class_qda, class_scda, class_plsda, class_svm, class_svm_t)

> par(mfrow = c(3, 1))

> comparison <- compare(classifierlist, plot = TRUE, measure = c("misclassification", "brier score", "average probability"))

> print(comparison)

**Table 5:** 

	misclassification	brier.score	average.probability
DLDA	0.06807692	0.13420913	0.9310332
LDA	0.04269231	0.07254283	0.9556106
QDA	0.24000000	0.34247861	0.7362778
scDA	0.01910256	0.03264544	0.9754012
pls_lda	0.01743590	0.02608426	0.9819003
svm	0.06076923	0.12077855	0.7872984
svm2	0.04461538	0.10296755	0.8014135

The tuned SVM labeled svm2 performs slightly better than it is untuned version, though the difference is not substantial. Compared with the performance of the other classifiers applied to this dataset, it seems that the additional complexity of the SVM does not pay out in a setting where even simple methods perform well.

#### 3.2.2 Running times

Table 6 shows the running times corresponding to the classifiers and variable selection methods outlined above, where * indicates that other programming languages than R are called, e.g. C/C++, Fortran. All computations were executed with a Pentium IV workstation, 2.8 Ghz, 1 GB main memory. The operating system was Windows XP.

**Table 6:** Running times.

**Variable selection methods**		
Method		Running time per learningset
Multiclass F-Test		3.1 s
Krusal-Wallis test		3.5 s
Limma*		0.16s
Random Forest^†,*^		4.1 s

**Classification methods**		
Method	# variables	Running time per **50 **learningsets

DLDA	all (2308)	2.7 s
LDA	10	1.4 s
QDA	2	1.0 s
Partial Least Squares	all (2308)	4.2 s
Shrunken Centroids	all (2308)	2.8 s
SVM*	all (2308)	88s

Running times of the different variable selection and classification methods used in the real life example. †: 500 bootstrap trees per run.

## 4 Conclusion

CMA is a new user-friendly Bioconductor package for constructing and evaluating classifiers based on a high number of predictors in a unified framework. It was originally motivated by microarray-based classification, but can also be used for prediction based on other types of high-dimensional data such as, e.g. proteomic, metabolomic data, or signal data. CMA combines user-friendliness (simple and intuitive syntax, visualization tools) and methodological strength (especially in respect to variable selection and tuning procedures). We plan to further develop CMA and include additional features. Some potential extensions are outlined below.

In the context of clinical bioinformatics, researchers often focus their attention on the additional predictive value of high-dimensional molecular data given that good clinical predictors are already available. In this context, combined classifiers using both clinical and high-dimensional molecular data have been recently developed [18,46]. Such methods could be integrated into the CMA framework by defining an additional argument corresponding to (mandatory) clinical variables.

Another potential extension is the development of procedures for measuring the stability of classifiers, following the scheme of our Bioconductor package 'GeneSelector' [47] which implements resampling methods in the context of univariate ranking for the detection of differential expression. In our opinion, it is important to check the stability of predictive rules with respect to perturbations of the original data. This last aspect refers to the issue of 'noise discovery' and 'random findings' from microarray data [41,48]. In future research, one could also work on the inclusion of additional information about predictor variables in the form of gene ontologies or pathway maps as available from KEGG [49] or cMAP  with the intention to stabilize variable selection and to simultaneously select groups of predictors, in the vein of the so-called "gene set enrichment analysis" [50].

As kindly suggested by a reviewer, it could also be interesting to combine several classifiers into an ensemble. Such aggregated classifiers may be more robust and thus perform better than each single classifier. As a standardized interface to a large number of classifiers, CMA offers the opportunity to combine their results with very little effort.

Lastly, CMA currently deals only with classification. The framework could be extended to other forms of high-dimensional regression, for instance high-dimensional survival analysis [51-54].

In conclusion, we would like to outline in which situations CMA may help and warn against potential wrong use. CMA provides a unified interface to a large number of classifiers and allows a fair evaluation and comparison of the considered methods. Hence, CMA is a step towards reproducibility and standardization of research in the field of microarray-based outcome prediction. In particular, CMA users do not favor a given method or overestimate prediction accuracy due to wrong variable selection/tuning schemes. However, they should be cautious while interpreting and presenting their results. Trying all available classifiers successively and reporting only the best results would be a wrong approach [6] potentially leading to severe "optimistic bias". In this spirit, Ioannidis [41] points out that many results obtained with microarray data are nothing but "noise discovery" and Daumer et al [55] recommend to try to validate findings in an independent data set, whenever possible and feasible. In summary, instead of fishing for low prediction errors using all available methods, one should rather report all the obtained results or validate the best classifier using independent fresh validation data. Note that both procedures can be performed using CMA.

## 5 Availability and requirements

• Project name: CMA

• Project homepage: 

• Operating system: Windows, Linux, Mac

• Programming language: R

• Other requirements: Installation of the R software for statistical computing, release 2.7.0 or higher. For full functionality, the add-on packages 'MASS', 'class', 'nnet', 'glmpath', 'e1071', 'randomForest', 'plsgenomics', 'gbm', 'mgcv', 'corpcor', 'limma' are also required.

• License: None for usage

• Any restrictions to use by non-academics: None

## 6 Authors' contributions

MS implemented the CMA package and wrote the manuscript. ALB had the initial idea and supervised the project. ALB and MD contributed to the concept and to the manuscript.
